# Lower Extremity Salvage in a Diabetic Patient With Cutaneous Mucormycosis and COVID-19 After Open Patella Fracture

**DOI:** 10.31486/toj.21.0099

**Published:** 2022

**Authors:** Danny A. S. Hammoudi, Malika M. Morar, Anna Garbuzov, Daniel Urias, Kristopher M. Katira

**Affiliations:** ^1^ The University of Queensland Medical School, Ochsner Clinical School, New Orleans, LA; ^2^ Department of Plastic Surgery, Intermountain Medical Center, Murray, UT; ^3^ Department of Plastic Surgery, Tulane University, New Orleans, LA

**Keywords:** *COVID-19*, *diabetes mellitus*, *extremities*, *free tissue flaps*, *mucormycosis*, *patella*, *reconstructive surgical procedures*

## Abstract

**Background:** Cutaneous mucormycosis, while less common than sinonasal or pulmonary infections, can cause widespread tissue necrosis after seemingly innocuous encounters. The most common location of cutaneous mucormycosis is the extremities, and extensive infection has been reported after trauma or orthopedic procedures.

**Case Report:** A 60-year-old female with poorly controlled type 2 diabetes mellitus sustained an open patella fracture after a fall. She underwent washout and internal fixation with cannulated screws and cable tension band wiring. The patient's recovery was complicated by asymptomatic coronavirus disease 2019 (COVID-19) infection and repeated wound dehiscence, with growth of *Mucor* species initially presumed to be a contaminant. Despite serial washout and debridement, repeat dehiscence and patella exposure were noted. Free tissue transfer to the genicular vessels was selected for coverage of the extensor tendon, patella, and fracture line. In repeat skin cultures, *Mucor indicus* and *Staphylococcus epidermidis* grew from the wound. Topical voriconazole and a 6-week course of intravenous isavuconazole and oral doxycycline were started when the *Mucor* cultures were identified.

**Conclusion:** This case highlights an approach to an indolent mucormycosis infection in the skin over a patella fracture in a patient with poorly controlled diabetes mellitus, including the sequence of surgical care, debridement, and selection of antimicrobials. Major amputation and orthopedic revision were avoided. This patient also underwent successful free tissue transfer after testing positive for COVID-19.

## INTRODUCTION

Mucormycosis is a rare but serious opportunistic fungal infection caused by the mold mucormycetes. Immunocompromised individuals, including those with poorly controlled diabetes mellitus, are vulnerable to this infection, although mucormycosis can also affect immunocompetent hosts.^1,2^ Rhinocerebral mucormycosis is perhaps the most well-known disease process involving mucormycetes, characterized by rapidly progressive necrotizing infections of the sinuses that are associated with high mortality. Most of the surgical literature on mucormycosis focuses on the morbidity and mortality of rhinocerebral infections in immunocompromised patients, although the cutaneous variety is also a surgical and medical emergency because of its fulminant presentation.^2,3^ As such, mucormycosis infections of the skin should not be overlooked in the differential diagnosis of delayed healing in the diabetic or immunocompromised patient.^2^

Cutaneous mucormycosis is only the third most common subtype of this invasive fungal infection.^1,2^ The lesions can appear as macules or papules.^1,2,4^ While the course of mucormycosis skin infections is usually indolent, necrotizing processes have been described, including life- and limb-threatening scenarios.^4,5^ The most common risk factor, which has been implicated in up to 88% of cases, is penetrating trauma, with soil most commonly serving as the source of inoculation.^1,2^ Additional risk factors include burns, surgery, persistent maceration, and subcutaneous or intramuscular injections. Cutaneous mucormycosis may also arise as a nosocomial infection.^1,2,6^ The cornerstone of treatment for any manifestation of cutaneous mucormycosis is early surgical debridement and antifungals.^3,7^ When critical structures are exposed in such wounds, reconstructive solutions such as pedicled or free flaps may be required for coverage.^3^ Potent antifungals, such as amphotericin B, have historically been used to treat these infections but have poor side effect profiles involving multiple organ systems.^7^ This side effect profile has led to reports of new pharmacologic therapies, in particular triazoles and topical amphotericin B.^7^

The following case illustrates a multidisciplinary approach to addressing cutaneous mucormycosis in a patient who sustained an open patella fracture. Hardware preservation, fracture healing, and avoidance of amputation and major orthopedic revision were achieved.

## CASE REPORT

**Figure 1.**
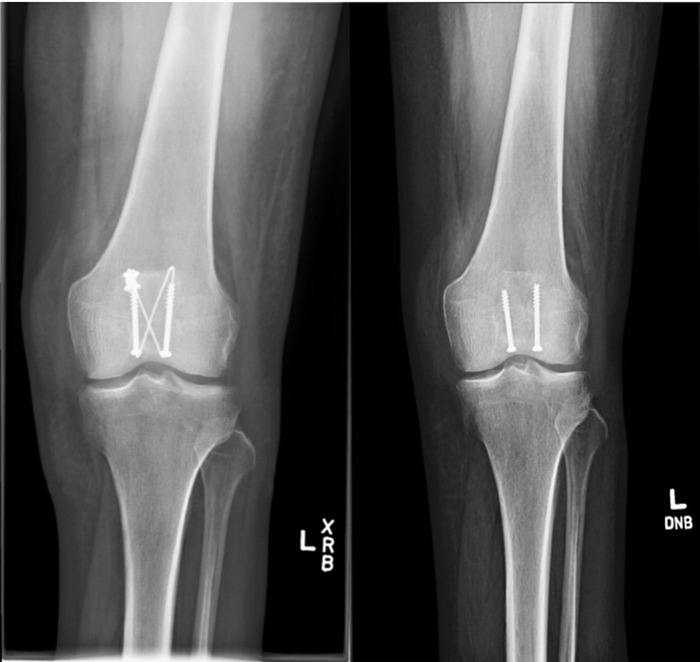
Cable screw construct for internal fixation of a transverse patella fracture before and after wire removal.

**Figure 2.**
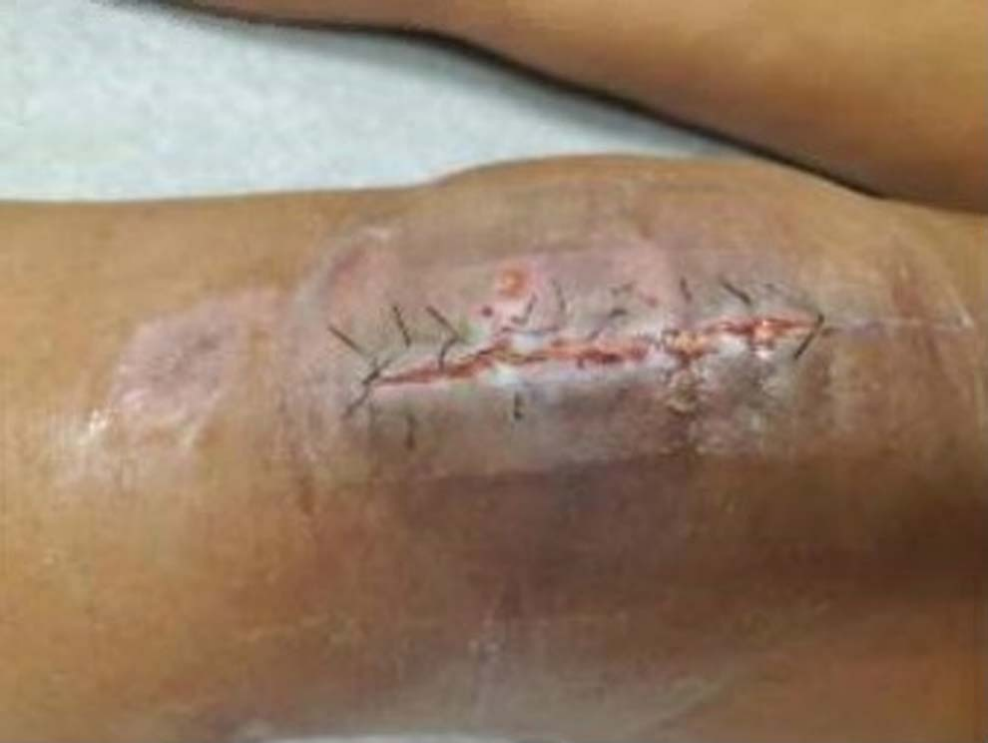
Incisional dehiscence after initial open reduction and internal fixation.

A 60-year-old female with poorly controlled type 2 diabetes mellitus (HbA1c 8.4%) on insulin without evidence of complications sustained an open patella fracture after a fall from standing onto moss-covered ground. She underwent washout and internal fixation with cannulated screws and cable tension band wiring (Figure 1).^8,9^ The patient had no acute postoperative complications; however, 6 weeks after initial presentation, she developed wound dehiscence (Figure 2) that was treated with washout and debridement followed by primary closure. Intraoperatively, the cables were exposed and removed, but the screws were not visible, and the decision was made to leave them in place as the relative risk of infection was considered low compared to the risk of patellar nonunion. Per recommendations from the orthopedic and infectious disease teams, she underwent a 6-week course of intravenous (IV) cefepime 2 g every 8 hours and vancomycin with a trough goal of 15-20 μg/mL for suspected infected hardware. After discharge, bacterial cultures collected in the operating room demonstrated rare growth of *Mucor* species, which was presumed to be a contaminant.

**Figure 3.**
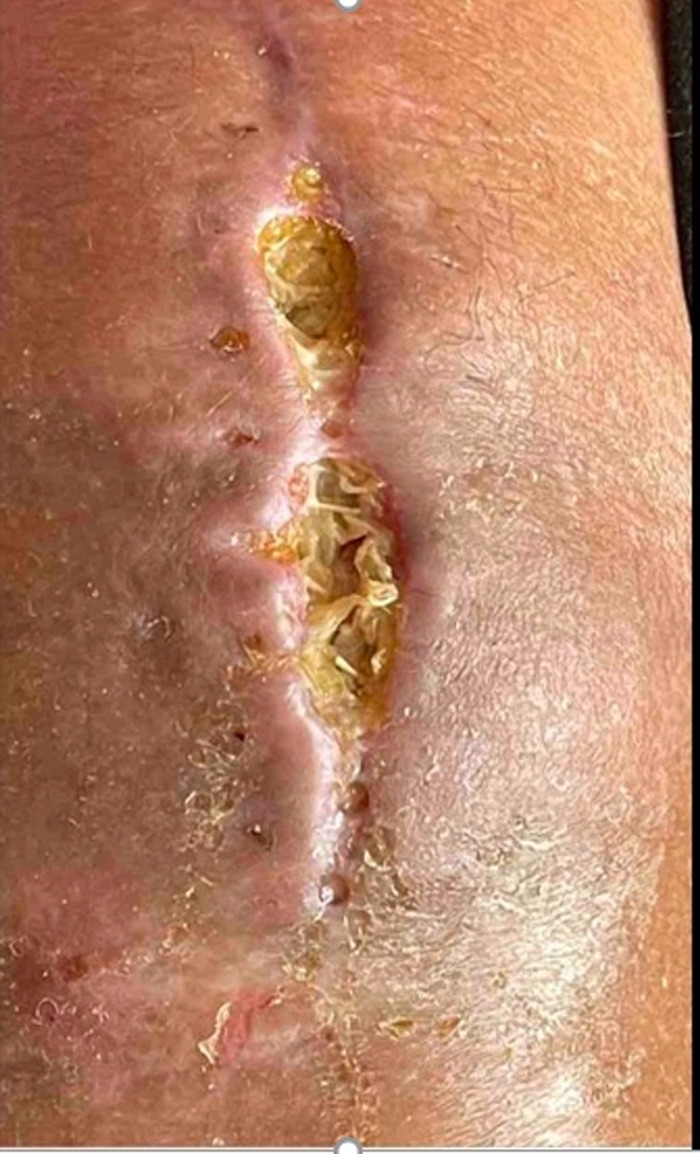
Repeat dehiscence after washout and wire removal.

**Figure 4.**
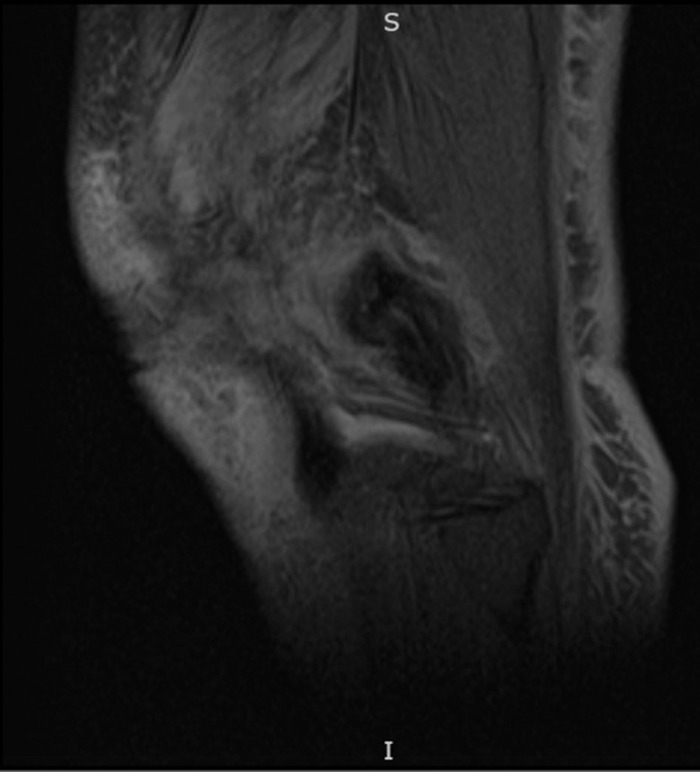
Cellulitis noted on magnetic resonance imaging corresponds with the wound shown in Figure 3.

Eight weeks after initial presentation, the patient tested positive for coronavirus disease 2019 (COVID-19). The patient was at home on a 2-week quarantine and was monitored virtually; she did not develop symptoms associated with the COVID-19 infection and did not require any treatment. A second dehiscence (Figure 3) occurred at this time, unrelated to the COVID-19 infection. Magnetic resonance imaging (MRI) revealed mild cellulitis circumferential to the knee joint, but the MRI showed no signs of deep muscle involvement or any bone marrow edema to suggest osteomyelitis (Figure 4).

Thirteen weeks after initial presentation, the patient had not responded to antibiotic therapy and was taken again for serial washout and debridement with 1 g of topical vancomycin powder placed in the wound and closure with localized tissue advancement. She was discharged after the procedure with instructions to resume vancomycin and cefepime infusions. At her clinic follow-up 1 week postoperatively, she was switched to oral doxycycline 100 mg twice daily per infectious disease for prophylaxis, as intraoperative cultures came back negative and she had completed a 7-week course of IV antibiotics.

**Figure 5.**
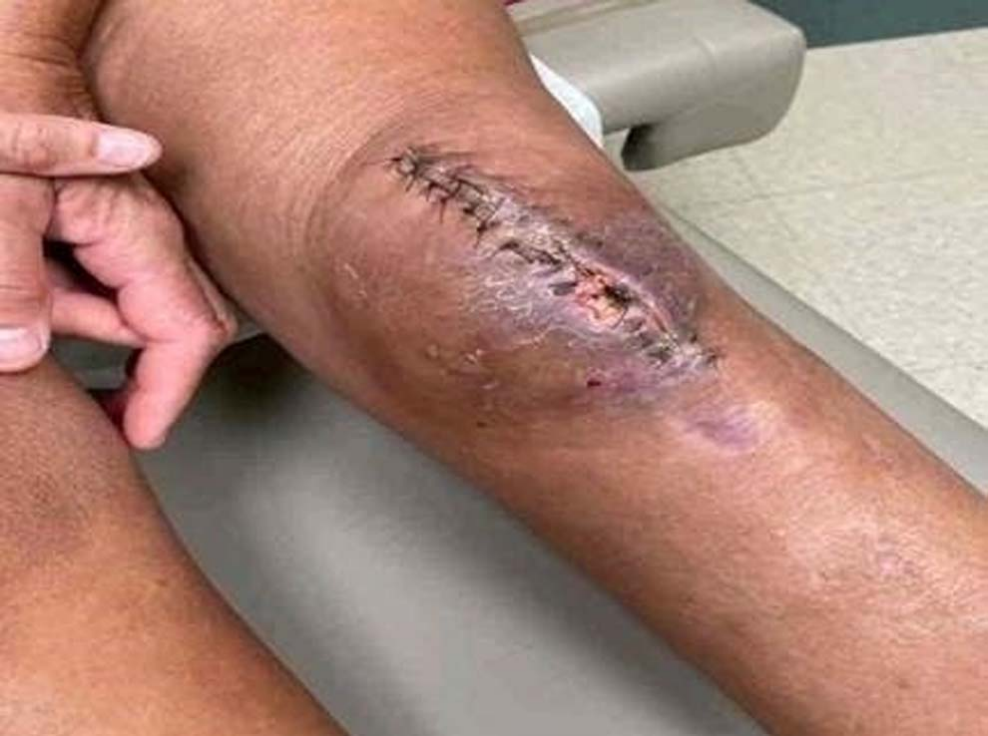
Patella exposure after fasciocutaneous flap elevation and complex closure. Wide skin discoloration surrounds the lower two-thirds of the knee incision.

**Figure 6.**
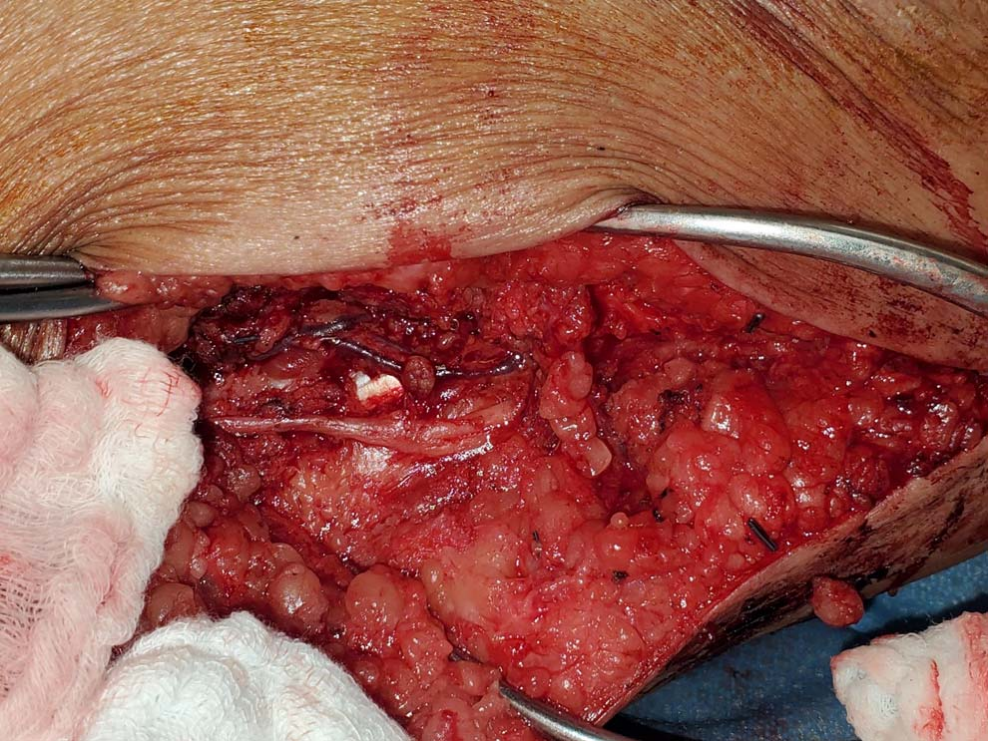
The genicular vessels were exposed along the medial thigh for free tissue transfer. The saphenous nerve was preserved.

**Figure 7.**
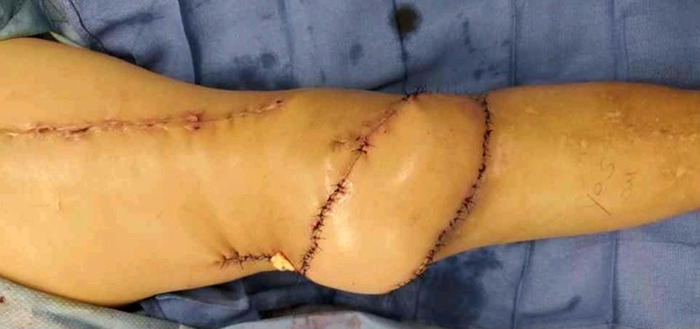
Anterolateral thigh free flap closure of knee wound after radical debridement of soft tissues.

Despite these efforts, a third dehiscence was noted at her 2-week clinic follow-up, now with patella exposure, requiring radical debridement of the violaceous knee skin (Figure 5). She was admitted to the hospital, and free tissue transfer to the genicular vessels was performed for coverage of the extensor tendon, patella, and fracture line (Figures 6 and 7). On postoperative days (POD) 1 to 3, the patient required an insulin drip and adjustment of long-acting insulin to treat episodes of hyperglycemia ranging from 200 to 370 mg/dL. She progressed appropriately and was transferred out of the intensive care unit. On POD 4, with abundance of precaution given previous *Mucor* growth, the patient was started on dual therapy with oral isavuconazole 186 mg daily and oral doxycycline 100 mg twice daily for 14 days. On POD 7, she started the dangling protocol and tolerated it well. On POD 9, cultures collected in the operating room again demonstrated growth of *Mucor* species. Fungal operative cultures took this amount of time to be reported as final. On POD 12, the patient met all milestones for discharge and was discharged home with home health. The patient received 1 month of aspirin 81 mg and enoxaparin 40 mg daily until she was no longer bedbound.

**Figure 8.**
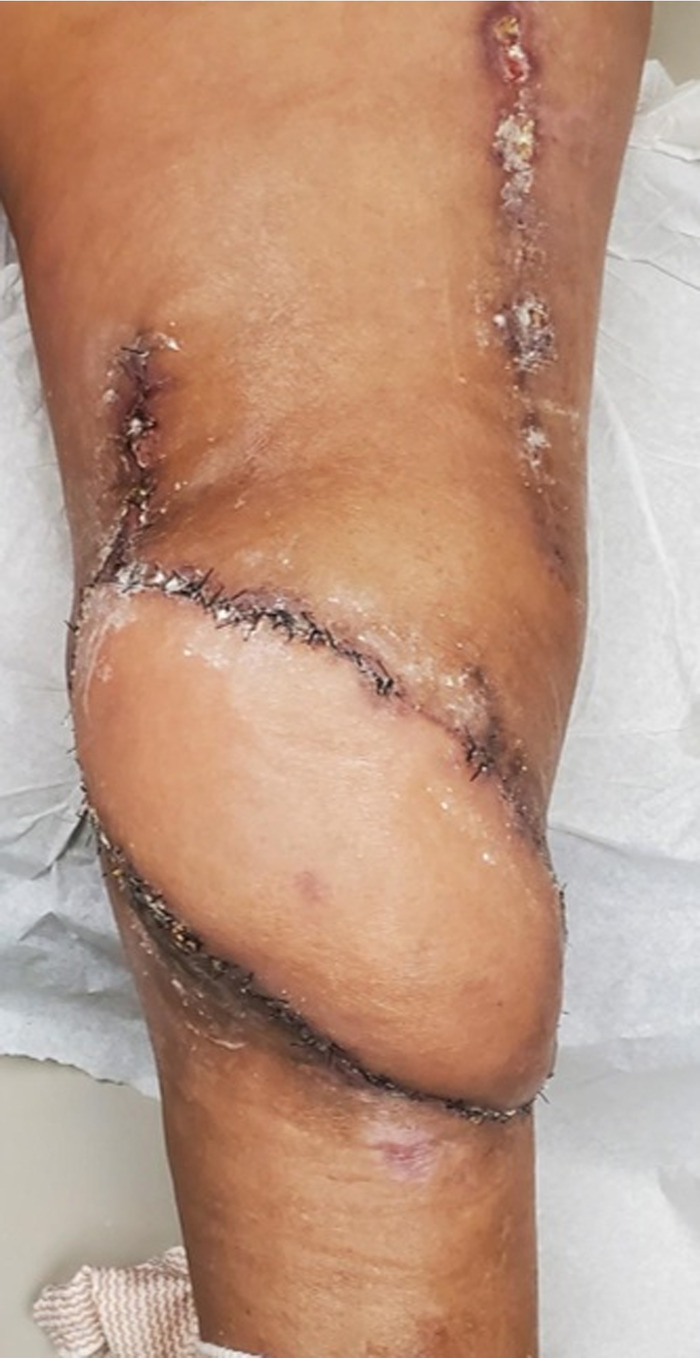
Topical antifungals were placed along the flap incision lines to facilitate healing in the postoperative period.

**Figure 9.**
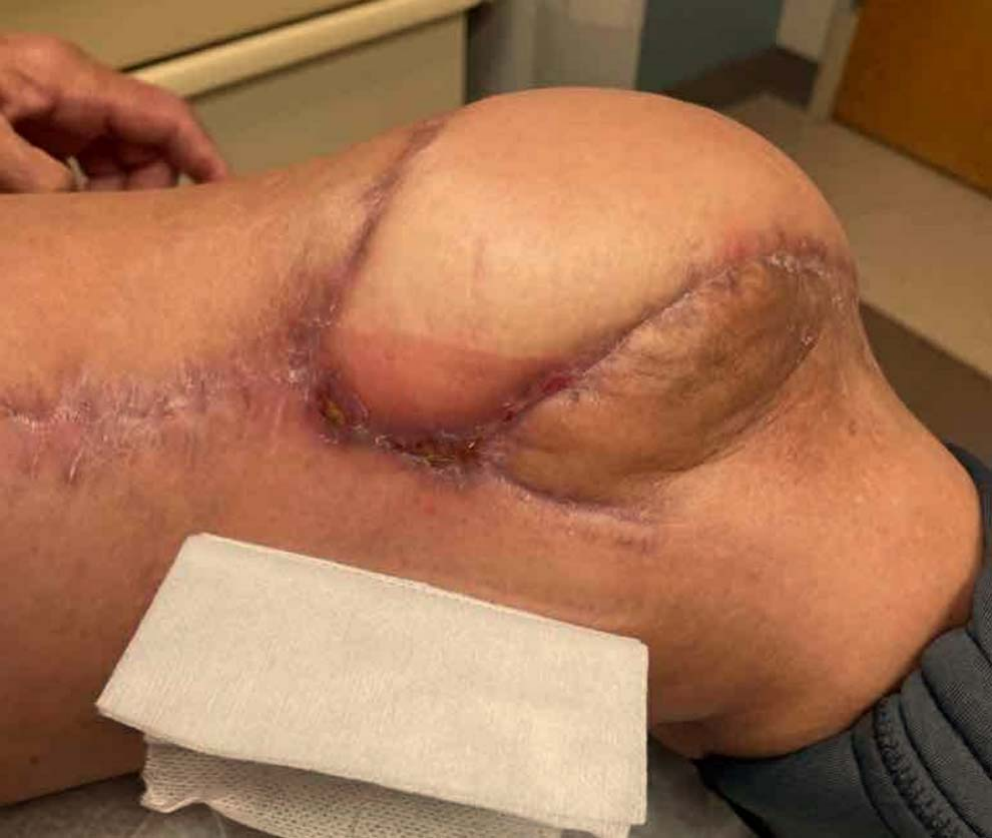
The flap was healing well 4 months after transfer. The patient demonstrated knee range of motion and used compression therapy for edema control.

At 2-week follow-up, the flap was viable, but healing was delayed with notable dehiscence along the suture lines. The patient was taken for a fourth washout and debridement of the flap the following week. *Mucor* species was noted from the skin cultures, and *Staphylococcus epidermidis* grew from the wound. The *Mucor* sample was sent for fungal culture and sensitivity. A 6-week course of oral isavuconazole 372 mg daily and oral doxycycline 100 mg twice daily was started when the *Mucor* cultures resulted from this latest washout. After 4 weeks, the fungal culture identified *Mucor indicus,* and the patient was switched to a 6-month course of oral posaconazole 100 mg 3 times daily based on susceptibility testing. A 1-month course of topical voriconazole was added to her therapy when marginal necrosis was noted around the flap in the postoperative period (Figure 8). After 6 weeks, all sutures were removed, and she started bearing weight 6 weeks postoperatively with a brace (Figure 9). She completed a 6-month course of oral posaconazole 100 mg 3 times daily.

## DISCUSSION

This case highlights the difficulty of recognizing a cutaneous mucormycetes infection. The mechanism of sustaining a wound after a fall onto soil, poorly controlled diabetes mellitus, and the discoloration and ulceration seen along the patient's incision line are characteristic of this infection. Repeated wound healing difficulties despite wire removal, customary empiric antimicrobial therapy, and meticulous local soft tissue closures indicate unusually poor healing reserves. In any patient with this scenario, possible etiologies include peripheral vascular disease, diabetic hyperglycemia, active or chronic nicotine use, inadequate immobilization, atypical infection, or hardware infection. Given the continued strength of the hardware construct and stable inflammatory markers, hardware infection was deemed unlikely. *Mucor* growth in multiple separate sets of cultures ultimately suggested a fungal etiology of delayed healing. Multiple cultures were required to tailor antifungal therapy, highlighting the importance of prolonged therapy and the need for extended antibiotics to eradicate this infection. When fungal skin infections are suspected, skin specimens should be sent to pathology for tissue evaluation in addition to the standard microbiology studies to confirm a diagnosis of mucormycosis.^2,3^ Our patient had an indolent course of cutaneous mucormycosis and not the necrotizing variety.^10^ The need for serial debridements and washouts in this case highlights the need to make a prompt diagnosis, as well as the importance of keeping fungal infection in the differential diagnosis of infection or delayed wound healing. Specific treatment strategies for mucormycosis include aggressive debridement of necrotic tissues, stabilization of glycemic control and nutrition, culture-directed antimicrobials, and well-vascularized soft tissue coverage.

Orthopedic care for this patient's open transverse patella fracture was critical for salvage. Percutaneous cannulated screw fixation with tension band wiring permitted stabilization of the knee extensor mechanism.^11^ Wire removal allowed for elimination of a potential cause of delayed healing after the first episode of dehiscence. Consultation with plastic surgery was appropriate after attempting complex closure because the patella was not well covered. If bone necrosis, joint infections, or necrotizing soft tissue processes had been present, amputation or major revision may have been the only options in this case.

While the gastrocnemius flap is a reliable option for small to moderate peripatellar defects, larger wounds require free tissue transfer for coverage.^3,12^ The decision to proceed with free tissue transfer was further confirmed after interrogation of the genicular vessels revealed that these vessels were suitable. Free tissue transfer permitted a more controlled inset of soft tissues over the critical area of concern in the patella and distal thigh. A large-caliber artery and vein were present at the knee from the descending genicular system. At least 9 other cases of free tissue transfer to reconstruct soft tissue defects resulting from cutaneous mucormycosis infection have been published.^3^

Adding to the complexity of this case, this patient was in the convalescent phase of a COVID-19 infection during the last 2 surgeries. This patient was our first experience with free tissue transfer in a patient with COVID-19. As shown in this case and other reports, free tissue transfer is possible for select patients, despite the hypercoagulability associated with COVID-19.^13,14^ Our intention was to attempt conservative wound coverage strategies in the early stages of COVID-19 infection. One report of COVID-19 free tissue transfer recommends delaying reconstruction, and another report of lower extremity free tissue transfer in a patient with COVID-19 and a pilon fracture recommends an extended period of anticoagulation postoperatively, including 3 months of antiplatelet therapy and 6 months of therapeutic anticoagulation.^13,14^ Given the asymptomatic course of this patient's COVID-19 infection and the fact that she was more than 6 weeks past her initial positive result, a more conservative regimen consisting of 1 month of aspirin 81 mg and enoxaparin 40 mg daily chemoprophylaxis during bedrest was administered. Our patient's postoperative course was not complicated by sequelae of her COVID-19 infection, and the conservative regimen was successful in preventing any thromboembolic event. While infection with COVID-19 is an important consideration in patients undergoing free tissue transfer, our case suggests that the convalescent phase of a COVID-19 infection does not necessarily preclude definitive surgical management. More evidence is needed to determine the safety of free tissue transfer in the acute nonconvalescent stage of COVID-19 infection.

Antimicrobial selection was also critical to the final healing of the patient's soft tissues. Infectious disease narrowed her antimicrobials based on fungal culture sensitivities. Amphotericin B was avoided, given its toxicity profile, in favor of isavuconazole, which recent literature (2016, 2019) has proposed to be an effective first-line option for mucormycetes infection.^15,16^ Topical voriconazole was also administered along healing wound edges, with minimal systemic toxicity, and topical amphotericin B could also be considered.^17^ Extended IV antimicrobials may be indicated beyond initial soft tissue healing in orthopedic settings to avoid sequelae of deep space reinfections and fracture nonunions.^18^ Hyperbaric oxygen and tight glycemic control are also adjunctive medical strategies in scenarios of delayed healing supported in the literature for cutaneous mucormycosis.^19^

## CONCLUSION

Aggressive soft tissue debridement, antifungals, and combined orthopedic and plastic surgery intervention are critical to achieving limb salvage in a patient with cutaneous mucormycosis and an open fracture. Submission of skin cultures to pathology should be considered when fungal infections are suspected or should be ruled out. Antifungal empiric antimicrobial therapies should be considered in patients with risk factors for a fungal infection, including patients with poorly controlled diabetes mellitus, immunocompromised patients, and patients with a soil inoculation mechanism. Free tissue transfer should be considered as an option when radical debridement leaves a large soft tissue defect involving the superior part of the patella exposed. This case also highlights that free tissue transfer is possible in a patient with COVID-19. When possible, delay of a free tissue transfer procedure in patients with COVID-19 should be strongly considered to ensure that the patient is medically stable or in the convalescent phase of the disease, thus minimizing risk of hypercoagulability. Multispecialty care is critical to achieving limb salvage success in cases of cutaneous mucormycosis.

## Notes

*This article meets the Accreditation Council for Graduate Medical Education and the American Board of Medical Specialties Maintenance of Certification competencies for Patient Care and Medical Knowledge.*

## References

[1] Petrikkos G, Skiada A, Lortholary O, Roilides E, Walsh TJ, Kontoyiannis DP. Epidemiology and clinical manifestations of mucormycosis. Clin Infect Dis. 2012;54suppl 1:S23-S34. doi: 10.1093/cid/cir866 22247442

[2] Coerdt KM, Zolper EG, Starr AG, Fan KL, Attinger CE, Evans KK. Cutaneous mucormycosis of the lower extremity leading amputation in two diabetic patients. Arch Plast Surg. 2021;48(2):231-236. doi: 10.5999/aps.2020.00549 33657778 PMC8007453

[3] Reinbold C, Derder M, Hivelin M, Ozil C, Al Hindi A, Lantieri L. Using free flaps for reconstruction during infections by mucormycosis: a case report and a structured review of the literature. Ann Chir Plast Esthet. 2016;61(2):153-161. doi: 10.1016/j.anplas.2015.05.006 26113355

[4] Jevalikar G, Sudhanshu S, Mahendru S, Sarma S, Farooqui KJ, Mithal A. Cutaneous mucormycosis as a presenting feature of type 1 diabetes in a boy – case report and review of the literature. J Pediatr Endocrinol Metab. 2018;31(6):689-692. doi: 10.1515/jpem-2017-0404 29672274

[5] Arnáiz-García ME, Alonso-Peña D, González-Vela Mdel C, García-Palomo JD, Sanz-Giménez-Rico JR, Arnáiz-García AM. Cutaneous mucormycosis: report of five cases and review of the literature. J Plast Reconstr Aesthet Surg. 2009;62(11):e434-e441. doi: 10.1016/j.bjps.2008.04.040 18684680

[6] Prakash H, Chakrabarti A. Global epidemiology of mucormycosis. J Fungi (Basel). 2019;5(1):26. doi: 10.3390/jof5010026 PMC6462913 30901907

[7] Greenberg RN, Scott LJ, Vaughn HH, Ribes JA. Zygomycosis (mucormycosis): emerging clinical importance and new treatments. Curr Opin Infect Dis. 2004;17(6):517-525. doi: 10.1097/00001432-200412000-00003 15640705

[8] Tian Y, Zhou F, Ji H, Zhang Z, Guo Y. Cannulated screw and cable are superior to modified tension band in the treatment of transverse patella fractures. Clin Orthop Relat Res. 2011;469(12):3429-3435. doi: 10.1007/s11999-011-1913-z 21573937 PMC3210283

[9] Yang TY, Huang TW, Chuang PY, Huang KC. Treatment of displaced transverse fractures of the patella: modified tension band wiring technique with or without augmented circumferential cerclage wire fixation. BMC Musculoskelet Disord. 2018;19(1):167. doi: 10.1186/s12891-018-2092-9 29793461 PMC5968519

[10] Telich-Tarriba JE, Pérez-Ortíz AC, Telich-Vidal J. Necrotizing fasciitis caused by cutaneous mucormycosis: a case report. Article in Spanish. Cir Cir. 2012;80(5):462-465. 23351453

[11] Cho JH. Percutaneous cannulated screws with tension band wiring technique in patella fractures. Knee Surg Relat Res. 2013;25(4):215-219. doi: 10.5792/ksrr.2013.25.4.215 24369000 PMC3867615

[12] Tiourin E, Kanack M, Ng W, Leis A. Mucor osteomyelitis of the distal radius necessitating ulnocarpal fusion. Cureus. 2021;13(1):e12813. doi: 10.7759/cureus.12813 33500870 PMC7817546

[13] Nassar AH, Maselli AM, Dowlatshahi AS. Microvascular free tissue transfer in the setting of COVID-19 associated coagulopathy: a case report. Orthoplastic Surg. 2021;4:20-22. doi: 10.1016/j.orthop.2021.02.002

[14] Kanatas A, Hart P, Mücke T. Hypercoagulability following COVID-19 infection: at what stage is it safe to do a free flap? Br J Oral Maxillofac Surg. 2020;58(10):e232-e233. doi: 10.1016/j.bjoms.2020.08.092 33008624 PMC7439826

[15] Marty FM, Ostrosky-Zeichner L, Cornely OA, Isavuconazole treatment for mucormycosis: a single-arm open-label trial and case-control analysis. Lancet Infect Dis. 2016;16(7):828-837. doi: 10.1016/s1473-3099(16)00071-2 26969258

[16] Zuglian G, Ripamonti D, Tebaldi A, Rizzi M. Cutaneous mucormycosis by *Rhizopus arrhizus* treated with isavuconazole as first line therapy: a case report. Med Mycol Case Rep. 2019;26:42-43. doi: 10.1016/j.mmcr.2019.10.002 31667060 PMC6812037

[17] Konigsberg MW, Wu CH, Strauch RJ. Topical treatment for cutaneous mucormycosis of the upper extremity. J Hand Surg Am. 2020;45(12):1189.e1-1189.e5. doi: 10.1016/j.jhsa.2020.01.015 32216989

[18] Metsemakers WJ, Kuehl R, Moriarty TF, Infection after fracture fixation: current surgical and microbiological concepts. Injury. 2018;49(3):511-522. doi: 10.1016/j.injury.2016.09.019 27639601

[19] Bentur Y, Shupak A, Ramon Y, Hyperbaric oxygen therapy for cutaneous/soft-tissue zygomycosis complicating diabetes mellitus. Plast Reconstr Surg. 1998;102(3):822-824. doi: 10.1097/00006534-199809030-00030 9727450

